# A Comparison Study of Fatigue Behavior of Hard and Soft Piezoelectric Single Crystal Macro-Fiber Composites for Vibration Energy Harvesting

**DOI:** 10.3390/s19092196

**Published:** 2019-05-13

**Authors:** Mahesh Peddigari, Ga-Yeon Kim, Chan Hee Park, Yuho Min, Jong-Woo Kim, Cheol-Woo Ahn, Jong-Jin Choi, Byung-Dong Hahn, Joon-Hwan Choi, Dong-Soo Park, Jae-Keun Hong, Jong-Taek Yeom, Kwi-Il Park, Dae-Yong Jeong, Woon-Ha Yoon, Jungho Ryu, Geon-Tae Hwang

**Affiliations:** 1 Korea Institute of Materials Science (KIMS), Changwon 51508, Korea; mahesh.p@kims.re.kr (M.P.); gayeon713@kims.re.kr (G.-Y.K.); chpark@kims.re.kr (C.H.P.); yuhomin@kims.re.kr (Y.M.); jwk@kims.re.kr (J.-W.K.); cheoruahn@kims.re.kr (C.-W.A.); finaljin@kims.re.kr (J.-J.C.); cera72@kims.re.kr (B.-D.H.); jchoi@kims.re.kr (J.-H.C.); pds1590@kims.re.kr (D.-S.P.); jkhong@kims.re.kr (J.-K.H.); yjt96@kims.re.kr (J.-T.Y.); zeppelin@kims.re.kr (W.-H.Y.); 2 School of Materials Science and Engineering, Kyungpook National University, Daegu 41566, Korea; kipark@knu.ac.kr; 3 Department of Materials Science and Engineering, Inha University, Incheon 22212, Korea; dyjeong@inha.ac.kr; 4 School of Materials Science and Engineering, Yeungnam University, Gyeongsan 38541, Korea

**Keywords:** energy harvesting, piezoelectric single crystal, long-term stability

## Abstract

Designing a piezoelectric energy harvester (PEH) with high power density and high fatigue resistance is essential for the successful replacement of the currently using batteries in structural health monitoring (SHM) systems. Among the various designs, the PEH comprising of a cantilever structure as a passive layer and piezoelectric single crystal-based fiber composites (SFC) as an active layer showed excellent performance due to its high electromechanical properties and dynamic flexibilities that are suitable for low frequency vibrations. In the present study, an effort was made to investigate the reliable performance of hard and soft SFC based PEHs. The base acceleration of both PEHs is held at 7 m/s^2^ and the frequency of excitation is tuned to their resonant frequency (*f*_r_) and then the output power (*P*_rms_) is monitored for 10^7^ fatigue cycles. The effect of fatigue cycles on the output voltage, vibration displacement, dielectric, and ferroelectric properties of PEHs was analyzed. It was noticed that fatigue-induced performance degradation is more prominent in soft SFC-based PEH (SS-PEH) than in hard SFC-based PEH (HS-PEH). The HS-PEH showed a slight degradation in the output power due to a shift in *f*_r_, however, no degradation in the maximum power was noticed, in fact, dielectric and ferroelectric properties were improved even after 10^7^ vibration cycles. In this context, the present study provides a pathway to consider the fatigue life of piezoelectric material for the designing of PEH to be used at resonant conditions for long-term operation.

## 1. Introduction

Over the past few years, research on vibration-based energy harvesting has increased tremendously by various groups across the globe. The main purpose of this research is to reduce the battery usage (or chemical waste) and to power the self-powered electronics by converting the ambient vibrations into electrical energy. Among the various vibration-based energy harvesting methods, including electromagnetic, electrostatic, piezoelectric, and triboelectric, the piezoelectric energy harvesting (PEH) is promising due to their ease of fabrication, structural integrity, high output power density and high conversion efficiency [1,2,3,4]. Among the various PEH configurations that have been examined for the efficient low frequency vibration energy scavenging, the cantilever based PEH is exceptional due to its simple structure and high root strain levels, which usually operates in bending mode to induce the in-plane strain in the cantilever structure according to the Euler-Bernoulli equation [1,4]. In general, the cantilever based PEH comprises of three essential parts including the elastic cantilever beam, piezoelectric layer, and proof mass. Numerous efforts have been made to improve the energy harvesting performance by optimizing the PEH configuration utilizing the materials with various strengths, dimensions, and strain levels [5,6,7,8,9,10,11,12,13,14,15,16,17,18,19]. In most cases, the maximum power is achieved under higher excitation levels due to the larger stress induced strains at the root of the cantilever. However, the application of such higher excitation forces often leads to performance degradation in the cantilever based PEHs. Therefore, a prior knowledge of behavior of the integral components under various excitation conditions is essential for designing high performance devices and for selecting the suitable materials to be used in specific applications.

Regarding the materials selection, depending on the excitation stimuli (frequency and acceleration), two kinds of piezoelectric materials—soft- and hard-type materials—have been widely utilized in harvesting applications [20,21,22]. In general, soft type piezoelectric materials exhibit higher piezoelectric coefficients and elastic compliances, which are more suitable for off-resonance excitations, whereas the hard type piezoelectric materials generate larger output power under resonant excitations owing to their larger mechanical quality factor. In order to improve the output performance in off-resonance conditions, a number of efforts such as non-linear dynamics and frequency-up conversion techniques were employed [23,24,25,26,27]. Thus far, piezoelectric materials have been used as active materials in cantilever based PEHs in various forms including thin/thick ceramic patches (or wafers), polymers, active fiber composites (AFC), macro-fiber composites (MFC), or single crystal macro-fiber composites (SFC) [3,28], in which the MFC/SFC configuration offers high mechanical flexibility, stress-strain performance, endurance, and electromechanical properties. Although many reports are available on the energy harvesting performance of PEHs based on MFC/SFCs [29,30,31,32,33,34,35,36,37,38], the investigation on the reliability (fatigue behavior) of SFCs under continuous electromechanical cyclic loading over a long time is rare [39,40,41,42]. 

Henslee et al. investigated the life-time performance of soft-type MFC (M-2807-P1, PZT 5A1, Smart Material Co., Dresden, Germany) based PEH up to 2.5 × 10^8^ cycles by measuring the strain and tip displacements under the application of a particular voltage at different temperatures ranging between 15–145 °C [40]. They reported that the performance of MFC was decreased continuously above 50 °C. In another study, Deepesh et al. performed the fatigue test for soft-type MFC (M-2807-P2, PZT 5A1, Smart Material Co.) based PEH at different excitation conditions and noticed the severe degradation in the output voltage with a significant shift in the resonant frequency even below 0.5 × 10^6^ cycles [41]. They proposed that the 600 με is the safe strain amplitude for the reliable operation over longer times. In another study, Panduranga et al. [42] investigated the damage propagation and different stages of fatigue failures in the soft type MFC (PZT 5A) based PEHs under various excitation levels in terms of change in the voltage and impedance values. Similarly, many reports are available on the fatigue behavior of soft-type piezoelectric materials and have discussed the fatigue life time, crack nucleation, and propagations. However, there are no reports available on the comparative studies on the fatigue behavior of soft- and hard-type SFC/MFCs under cyclic mechanical loads. Therefore, the present study intended to investigate the reliability performance of soft and hard SFCs at resonance condition under a constant excitation force (below the elastic limit) up to 10^7^ vibration cycles. 

## 2. Experimental details

**Figure 1:**
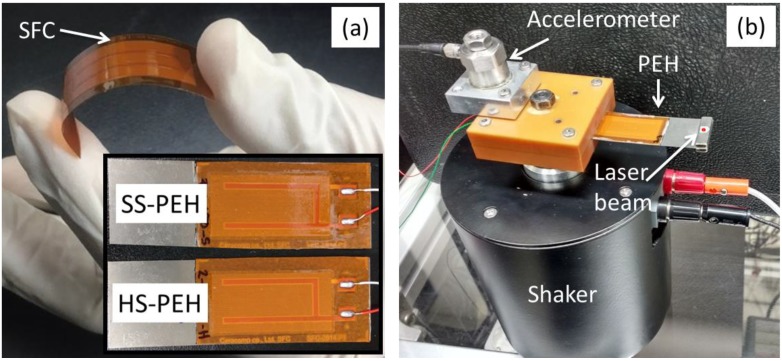
Photographs of (**a**) flexible single crystal-based fiber composites (SFC) and (**b**) experimental setup used for investigating the fatigue behavior of hard and soft SFC-based PEHs. The inset shows an image of soft and hard SFCs after attaching to the Ti-alloy elastic layer.

In the present study, two unimorph cantilever structured PEHs with two different Pb(Mg_1/3_Nb_2/3_)O_3_-Pb(Zr,Ti)O_3_ (PMN-PZT) based flexible SFCs fabricated by solid state grown method and electroded polyimide sheets lamination (Ceracomp. Co. Ltd., Korea) [35,36,37,38,43] were used as the active piezoelectric layers and a Ti alloy plate (elastic modulus of 55 GPa) was used as the passive elastic layer. Two NdFeB magnets (3 g) were used as a proof mass and attached at the free end of the cantilever. The Mn-doped PMN-PZT and W-doped PMN-PZT SFCs having the dimensions of 28 (L) × 14 (W) × 0.2 (T) mm^3^ were used as hard and soft piezoelectric materials, respectively (Figure 1a). The detailed manufacturing process of SFCs was reported in our previous study [32,33]. The SFCs were glued to a Ti alloy plate [60 (L) × 20 (W) × 0.25 (T) mm^3^] using an epoxy resin (3M^TM^ Scotch-Weld Epoxy Adhesive DP-460 EG) and then cured at 70 °C for 3 h. Further, the PEH was clamped with a rigid Bakelite holder and mounted on electromagnetic shaker for base excitation, as shown in Figure 1b. 

In order to investigate the reliable performance of hard and soft SFC-based PEHs, the specimens were tested up to 10^7^ fatigue cycles at a constant base acceleration of 7 m/s^2^ (~0.7 G) with 20~40 Hz, i.e., vicinity of resonance frequency of the PEHs using an electromagnetic shaker. A vibration controller system (Logtech Co., Korea) used to control the shaker, which is connected with a feedback acceleration sensor (PV-41, RION, Japan) and a Laser Doppler vibrometry (LDV; OFV-5000, Polytec, Germany). The vibration displacement of PEHs in time domain is measured using the LDV. For given excitation conditions, the harvested (root mean square, RMS) voltage (*V*_rms_) is measured across various load resistances (*R_L_*) embedded in a resistance decay box using a multi-channel multimeter (2700, Keithley, USA). Subsequently, the data were collected using National Instruments data acquisition systems and plotted the RMS power *P*_rms_ (= (*V*_rms_)^2^/*R_L_*) versus number vibration cycles using a home-made software. The time-dependent output voltage waveforms were captured using an oscilloscope (Wave surfer 44Xs-A, Lecroy, USA). The dielectric properties of SFCs were measured using an impedance analyzer (4294A, Agilent Technologies). The polarization-electric field hysteresis loops of SFCs were measured at 1 kHz using a ferroelectric tester (Precision LC-II, Radiant Technologies).

## 3. Results and Discussion

**Figure 2:**
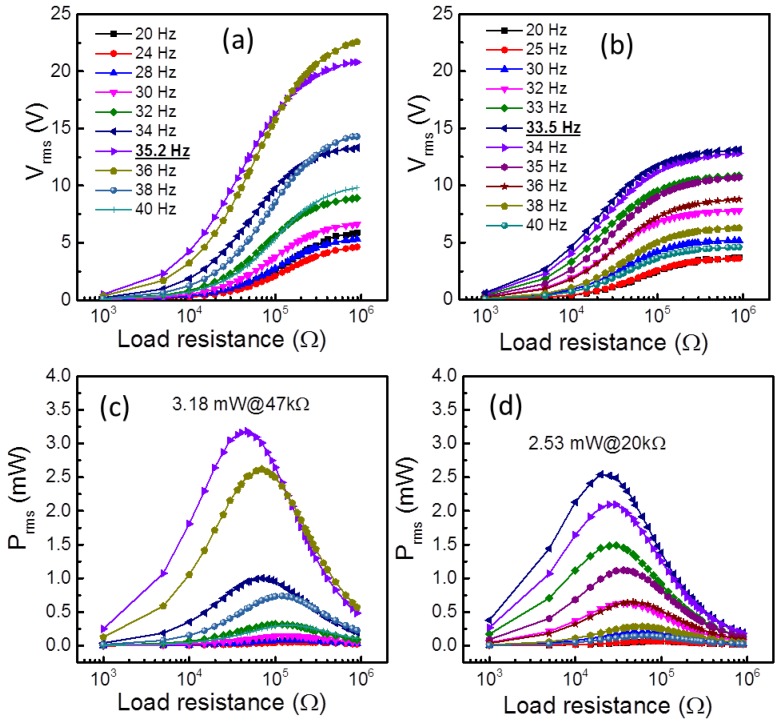
(**a**) and (**c**), (**b**) and (**d**) are the RMS voltage and RMS power curves for hard and soft SFC based piezoelectric energy harvesters (PEHs) measured as a function of load resistances at different excitation frequencies.

In order to know the resonant frequency (*f*_r_) and optimum load resistance (*R*_opt_) of both hard and soft SFC-based PEHs prior to test the fatigue behavior, the energy harvesting has been performed at an optimized base acceleration (7 m/s^2^) condition (Figure S1) under different excitation conditions by varying the frequency (20 Hz–40 Hz) and load resistances (1 kΩ–1000 kΩ). The *V*_rms_ response of both PEHs to the various load resistances (*R*_L_) for different excitation frequencies is depicted in Figure 2a,b) and the corresponding *P*_rms_ (= (*V*_rms_)^2^/*R*_L_) curves are shown in Figure 2c,d). Despite having identical geometry and acceleration excitation level, the PEHs exhibited different *f*_r_ and *P*_rms_ values due to the varied elastic compliance and electromechanical properties of SFCs (Table 1). The *f*_r_ values of the PEHs are identified from the maximum *P*_rms_ (or *P*_max_) values, which are found to be 35.2 Hz and 33.5 Hz for HS-PEH and SS-PEH, respectively. The HS-PEH exhibited a relatively larger *P*_max_ of 3.18 mW at 47 kΩ as compared to SS-PEH sample (2.53 mW at 20 kΩ) at the resonant condition. 

**Figure 3:**
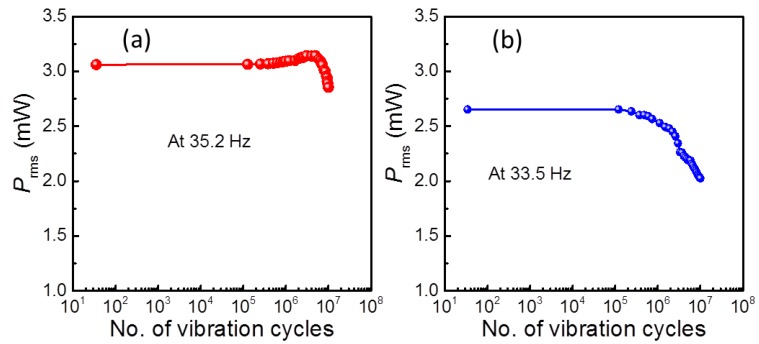
Variation in the *P*_rms_ response of (**a**) hard SFC-based PEH (HS-PEH) and (**b**) soft SFC-based PEH (SS-PEH) as a function of vibration cycles measured at 7 m/s^2^.

By using the optimized conditions *f*_r_ (35.2 Hz for HS-PEH and 33.5 Hz for SS-PEH) and *R*_opt_ (47 kΩ for HS-PEH and 20 kΩ for SS-PEH), the fatigue measurement has been performed at an acceleration of 7 m/s^2^ without interruption between the cycles. During the measurement, the harvested power *P*_rms_ was collected after every 1 h and plotted using software and the process was continued until the 10^7^-oscillation period was reached. The output response of PEHs to the number of vibration cycles is shown in Figure 3. It is observed that the output *P*_rms_ of HS-PEH is increased slightly with the vibration cycles up to 5 × 10^6^ cycles (Figure 3a), and decreased (~6.9%) further with increasing fatigue cycles, which might be due to the change in the *f*_r_. In the SS-PEH case, the output response deteriorated (~23.6 %) continuously with increasing the vibration cycles, as shown in Figure 3b. 

**Figure 4:**
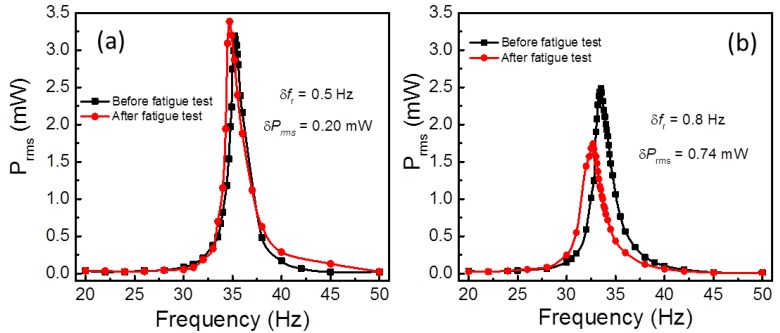
Comparison of power frequency response curves for (**a**) HS-PEH and (**b**) SS-PEHs measured at base acceleration of 7 m/s^2^.

**Figure 5:**
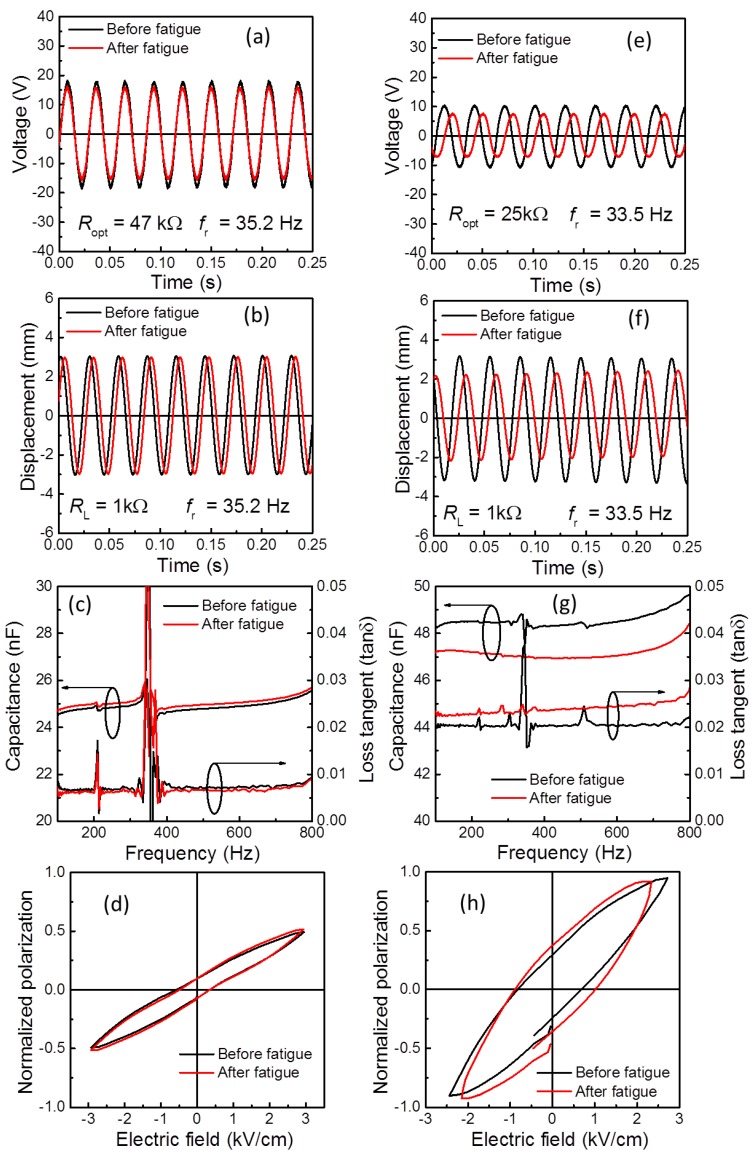
The comparison graphs of output voltage, vibration displacement, capacitance, and polarization changes for (**a**–**d**) HS-PEH and (**e**–**h**) SS-PEHs measured before and after fatigue test at 7 m/s^2^.

In general, the output response of the PEH under a cyclic mechanical load over a period of time can be related to various parameters such as polarization change due to re-orientation of defect dipoles, migration of charge carriers at domain wall, microstructural changes (crack propagation), and resonant frequency shift due to change in the stiffness of PEH. In order to understand the damage caused by the fatigue in both PEHs, we have compared some directly observable parameters such as *f*_r_, *V*_rms_, dielectric properties, polarization-electric field (*P-E*) loops, damping ratio, and vibration displacement (*d_v_*) before and after fatigue test in Figure 4 and Figure 5 and Table 2. 

The power frequency response curves for both PEHs before and after the fatigue test are presented in Figure 4a,b. It is noticed that the *f*_r_ for the HS-PEH decreased by 0.5 Hz in the span of 10^7^ vibration cycles, while the *P*_max_ and mechanical quality factor (*Q*_m_) were enhanced by about 0.20 mW and 0.06 mW, respectively. It can be understood that the abrupt drop in the power after 5 × 10^6^ cycles (Figure 3a) is a result from the shift of *f*_r_ rather than the hard SFC performance degradation. From Figure 4b, the SS-PEH displayed a significant frequency shift in *f*_r_ (0.8 Hz), reduction in *P*_max_ (0.74 mW) and *Q*_m_ (3.52), along with a change in *R*_opt_ from 20 kΩ to 25 kΩ after 10^7^ vibration cycles. In spite of a maximum *f*_r_ shift towards the low-frequency side, the SS-PEH exhibited reduced output power and *Q*_m_, indicating the performance deterioration is merely due to SFC material degradation.

As shown in Figure 5a,e, the output voltages of both PEHs measured at optimized conditions (*f*_r_ and *R*_opt_) show clear sinusoidal waves, which indicates that no cracks/damages were developed during the fatigue test (for post-test microstructural observation, see Figure S2). The HS-PEH show no significant reduction in the output voltage (Figure 5a) and vibration displacement (*d_v_*) (Figure 5b) even after 10^7^ fatigue cycles, while the SS-PEH show a substantial drop in the *V* and *d_v_* (Figure 5e,f) after fatigue test. In general, the maximum extractable power from a PEH depends on the base acceleration (*A*), resonance frequency (*f*_r_), and active (piezoelectric) layer volume (*V*piezo). In this context, the normalized volume power density (NVPD = *P*_max_/(*V*_piezo_. *f*_r_. *A*^2^)) was estimated for both PEHs [4]. The HS-PEH exhibited a relatively larger NVPD of 2.35 mW/cm^3^·Hz·g^2^ as compared to SS-PEH (1.97 mW/cm^3^·Hz·g^2^). Similar to the *P*_max_, the NVPD also improved for HS-PEH by 7.9% and decreased by 27.5% for SS-PEH after fatigue measurement (Table 2).

Similarly, Deepesh et al. observed that a severe reduction in the output voltage (with a 55% drop of initial voltage) of soft-type MFC within 2 × 10^6^ fatigue cycles at an acceleration of 6 m/s^2^ when the induced strain amplitudes slightly higher than 1000 με [41]. Further, they suggested that the strain amplitude of 600 με is an upper limit for reliable performance of soft-type MFC. In another study, Panduranga et al. also performed the fatigue measurement for soft-type MFC at different acceleration levels (4–6 m/s^2^) and stated that the MFC showed severe performance degradation at all the acceleration conditions and completely failed after 4.2 × 10^6^ fatigue cycles when MFC operated at 6 m/s^2^ with a maximum induced strain around 700 με [42]. In the present study, the maximum induced strains for the soft and hard SFCs used are slightly less than 300 με (Figure S3). Even though both SFCs showed crack-free behavior, the SS-PEH displayed severe performance degradation similar to the earlier reports, but the HS-PEH showed fatigue-free behavior with improved output power and mechanical quality factor.

Measuring the variation in piezoelectric properties of SFCs is a straightforward way to evaluate their fatigue ability, but since it is difficult, the dielectric and polarization properties were provided to supplement the piezoelectric properties. Figure 5c,g shows the dielectric properties such as capacitance (*C*) and loss tangent (*tanδ*) of PEHs measured in the frequency range of 100–800 Hz. The *C* of the HS-PEH is enhanced by around 0.7% (at 100 Hz) and reduced by 2.1% for SS-PEH, while the *tanδ* is reduced for HS-PEH and is increased for SS-PEH after 10^7^ fatigue cycles. From Figure 5d,h, the ferroelectric properties (measured at 1 kHz) follow a similar trend to the dielectric properties. The saturation polarization (*P*_S_) is slightly increased whereas the remanent polarization (*P*_r_) and coercive field (*E*_C_) remain constant for HS-PEH, while hysteresis loss is increased noticeably for SS-PEH after 10^7^ fatigue cycles. 

As seen in Figure 3, a slight reduction in the output power of HS-PEH is noticed during the fatigue measurement (due to a shift in *f*_r_), although the final output power at resonance condition is improved. The improved *P*_max_ and *Q*_m_ after fatigue measurement might be related to the stabilization (or change) of domain wall configuration [44]. In general, the acceptor based defect dipoles prefer to occupy the energetically favored sites in the lattice and form anisotropic centers locally or within the domain [45]. Though there are no (few) grain boundaries in single crystal fibers, it may be possible to increase the diffusion of acceptor-based defect dipoles into domain walls and orientate defect dipoles in the direction of spontaneous polarization with the fatigue cycles, which increases the domain wall pinning and the intensity of internal dipolar field. Thus, the stabilization of domain walls in HS-PEH leads to improved dielectric, ferroelectric, and piezoelectric properties along with *Q*_m_. In the case of SS-PEH, it is assumed that the reduction in the output power with increasing the fatigue cycles is related to the increased domain wall mobility, which is facilitated by the donor dopant defect dipoles under the cyclic mechanical force. As the domain wall mobility increases, more mechanical energy dissipates, which eventually increases the piezoelectric losses and lowers the *f*_r_ and *Q*_m_ values [46]. This phenomenon is evidenced from the increased dielectric and ferroelectric losses and reduced *Q*_m_ and output power.

The lack of significant performance degradation in the output power and mechanical quality factor with fatigue cycles indicates that hard-type SFCs exhibit good fatigue endurance and are reliable for long-term piezoelectric energy harvesting at resonant conditions.

## 4. Conclusions

In this study, we have investigated the fatigue reliability performance of hard and soft SFC-based PEHs at constant base acceleration for the use in long-term energy harvesting applications. After 10^7^ fatigue cycles, the degradation in the output power and shift in the resonant frequency of soft SFC-based PEH are higher than that of hard SFC-based PEH. Indeed, the HS-PEH showed significant improvements in the output power, capacitance, and saturation polarization even after long cyclic vibration life, whereas the SS-PEH underwent continuous deterioration. Therefore, this study can be helpful for researchers to consider the fatigue effect and to select the appropriate piezoelectric material for long-term operation in piezoelectric energy harvesting applications.

## Figures and Tables

**Table 1:** Various material properties of soft- and hard-type SFCs.

Parameter	Symbol	Hard-Type SFC (Mn-doped PMN-PZT)	Soft-Type SFC (W-doped PMN-PZT)	Units
Density	*ρ*	7800	7900	kg/m^3^
Volume	(*l* × *b* × *h*)	28 × 14 × 0.2	28 × 14 × 0.2	mm^3^
Dielectric constant	*ε*_33_	2250	3962	
Dielectric loss	tan*δ*	0.0018	0.005	
Mechanical loss	tan*θ*	0.003	0.010	
Electromechanical coupling factor	*k*_32_	0.697	0.726	
Piezoelectric charge constant	*d*_32_	−850	−1850	10^−12^ C/N
Elastic compliance	*s*_22_^E^	45.9	110.04	10^−12^ m^2^/N

**Table 2:** Changes in the energy harvesting characteristic parameters of PEHs after fatigue measurement.

Parameter	PEH Type	
	HS-PEH	SS-PEH
Δ*P*_rms_ (mW) (at initial *f*_r_)	−6.9%	−23.6%
Δ*P*_max_ (mW) (at final *f*_r_)	+0.20	−0.74
Δ*f*_r_ (Hz)	−0.5	−0.8
NVPD (mW/cm^3^·Hz·g^2^)	+7.9%	−27.5%
Δ*d_v_* (mm) (at initial *f*_r_)	−0.04	−1.20
Δ*C* (nF) (at 100 Hz)	+0.7%	−2.1%
*P*_S_ (μC/cm^2^)	+4.7%	−3.4%
Dielectric loss	−16.2%	+11.2%
Hysteresis loss	+4.6%	+20.6%
Mechanical loss (damping ratio)	−0.3%	+27.5%

## Supplementary Materials

The following are available online at https://www.mdpi.com/1424-8220/19/9/2196/s1, Figure S1: The acceleration dependent rms power measured for hard-type and soft-type SFCs. Figure S2: Surface micrographs of the (**a**) hard-type and (**b**) soft-type SFCs after 107 vibration cycles. Figure S3: Simulation results for SS-PEH, performed using COMSOL Multiphysics. The induced (**a**) stress and (**b**) strain distributions in the soft SFC under the resonance excitation condition.
