# Supplementary material for: A Comparison Study of Fatigue Behavior of Hard and Soft Piezoelectric Single Crystal Macro-Fiber Composites for Vibration Energy Harvesting
Source: Sensors (Basel). 2019 May 13;19(9):2196. doi: 10.3390/s19092196 (PMC6539409; doi:10.3390/s19092196)
Supplement: Supplementary file 1 [file sensors-19-02196-s001.pdf]

Supplementary Information

A Comparison Study of Fatigue Behavior of Hard and Soft Piezoelectric Single Crystal Macro-Fiber Composites for Vibration Energy Harvesting

Mahesh Peddigari[1], Ga-Yeon Kim[1], Chan Hee Park[1], Yuho Min[1], Jong-Woo Kim[1], Cheol-Woo Ahn[1], Jong-Jin Choi[1], Byung-Dong Hahn[1], Joon-Hwan Choi[1], Dong-Soo Park[1], Jae-Keun Hong[1], Jong-Taek Yeom[1], Kwi-Il Park[2], Dae-Yong Jeong[3], Woon-Ha Yoon[1], Jungho Ryu[4*], Geon-Tae Hwang[1*]

[1]Korea Institute of Materials Science (KIMS), Changwon 51508, Republic of Korea

[2]School of Materials Science and Engineering, Kyungpook National University, Daegu 41566, Republic of Korea

[3]Department of Materials Science and Engineering, Inha University, Incheon 22212, Republic of Korea.

[4]School of Materials Science and Engineering, Yeungnam University, Gyeongsan 38541, Republic of Korea

* Correspondence: jhryu@ynu.ac.kr, gthwang@kims.re.kr

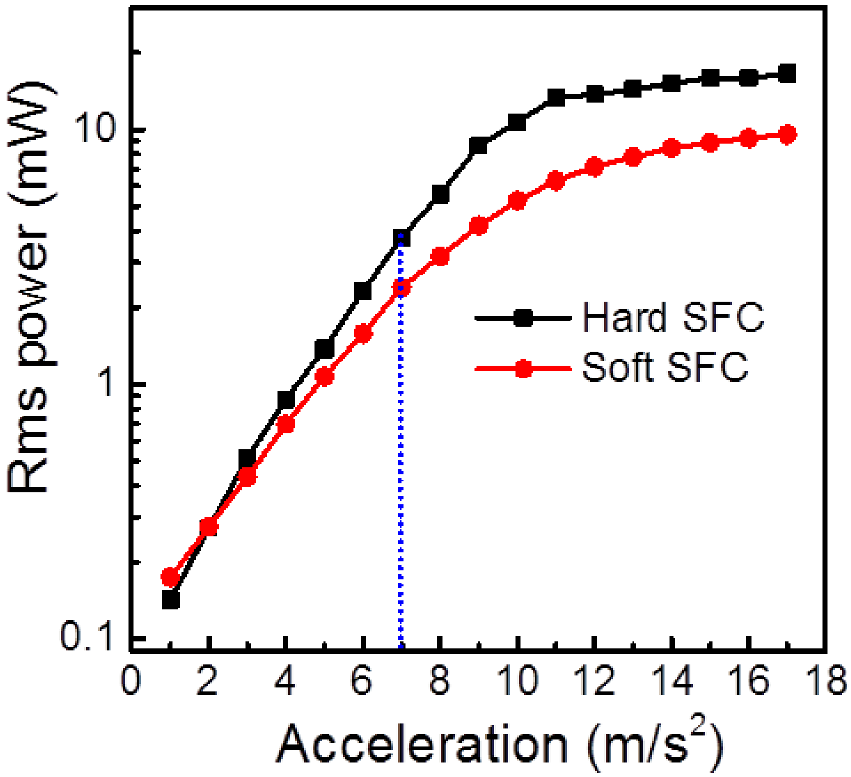

Figure S1. The acceleration dependent rms power measured for hard-type and soft-type SFCs.

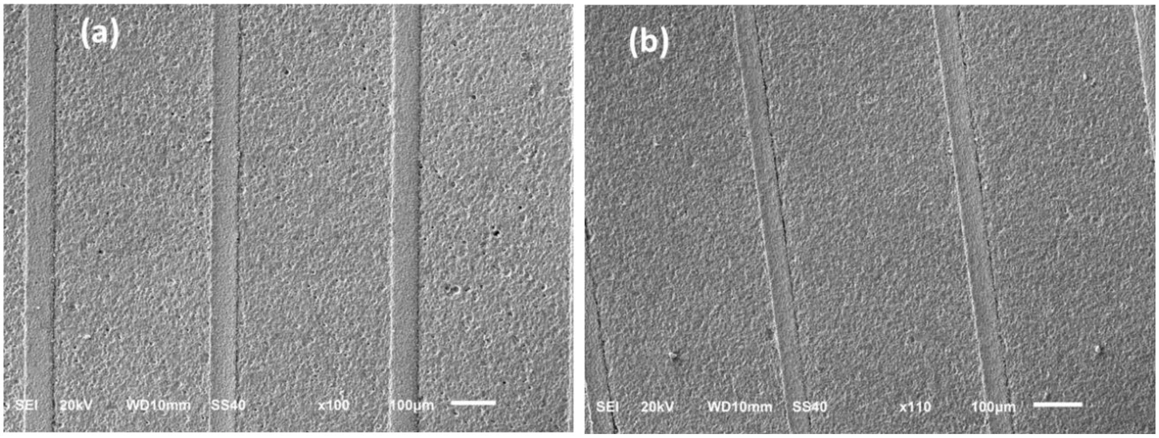

Figure S2. Surface micrographs of the (**a**) hard-type and (**b**) soft-type SFCs after 10^7 vibration cycles.

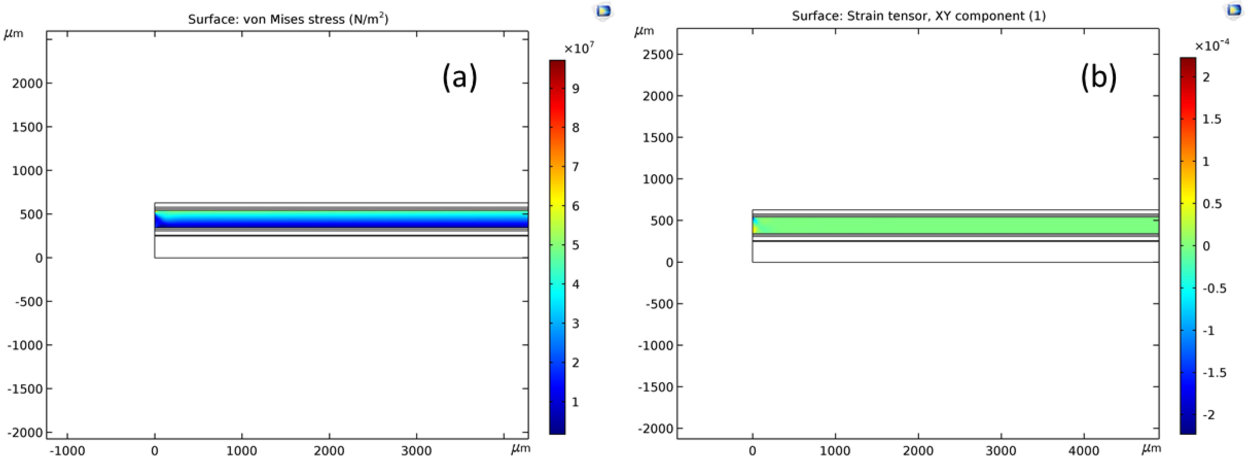

Figure S3. Simulation results for SS-PEH, performed using COMSOL Multiphysics. The induced (**a**) stress and (**b**) strain distributions in the soft SFC under the resonance excitation condition.
